# *Phyllostachys edulis* forest reduces atmospheric PM_2.5_ and PAHs on hazy days at suburban area

**DOI:** 10.1038/s41598-018-30298-9

**Published:** 2018-08-22

**Authors:** Yu Fang Bi, Fei Yan Guo, Liu Yang, Hao Zhong, An Ke Wang, Yu Kui Wang, Zhi Zhuang Wu, Xu Hua Du

**Affiliations:** 1 Key Laboratory of State Forestry Administration on Bamboo Resources and Utilization, China National Bamboo Research Center, State Forestry Administration, Hangzhou, 310012 Zhe Jiang China; 2 Zhejiang Forestry Product Testing Station, Hangzhou, 310023 China

## Abstract

This study is aim to illustrate *Phyllostachys edulis*’ role in affecting air quality under hazy day and solar day. *P. edulis* is a crucial plants growing well at suburban area at China Southern. In this manuscript, on 2 weather conditions (hazy day; solar day), changes in atmospheric particulate matter (PM), polycyclic aromatic hydrocarbons (PAHs), associated volatile organic compounds (VOCs), and PAHs in leaves and soils were measured, with PM-detection equipment and the GC-MC method, in a typical bamboo forest at suburban areas. The results showed that: (1) Bamboo forest decreased atmospheric PM_2.5_ and PM_10_ concentrations significantly by 20% and 15%, respectively, on the hazy day nightfall time, when they were times higher than that on any other time. Also, similar effects on atmospheric PAHs and VOCs were found. (2) Significant increases in PAHs of leaves and soil were found inside the forest on the hazy day. (3) Bamboo forest also reduced the atmospheric VOC concentrations, and changed the compounds of 10 VOCs present in the highest concentration list. Thus, bamboo forests strongly regulate atmospheric PM_2.5_ through capture or retention, for the changes in atmospheric VOCs and increase in PAHs of leaves and soil.

## Introduction

With the development of the economy, people demand a better living quality. However, the air quality decreases and hazy weather occurs frequently to affect human. The hazy day affected more than 17 provinces with1.43 million km^2^ and over 0.6 billion people at 2013 in China^1^. Particulate matter (PM), especially fine particulates with an aerodynamic diameter less than 2.5 mm (PM_2.5_) and 10 mm (PM_10_), is very important indicator for hazy day. With the high pollution air continuously diffused in China at the beginning of 2013, 5 strong haze pollutions occurred in Beijing-Tianjin-Hebei region. At the most serious time, PM2.5 broke through 600 μg/m^3^, PM1 broke through 300 μg/m^3^, and the concentration of organic matter, sulfate and nitrate in PM1 reached 160,70,40 μ/m^3 ^^2^. PM_2.5_ and PM_10_ can adversely affect human health, resulting in premature mortality, pulmonary inflammation and accelerated atherosclerosis, among other conditions^3,4^. PM_2.5_ can easily pass through the nose and mouth, then penetrate the lungs, and subsequently cause a range of effects on humans, such as impaired lung function and the loss of hemoglobin oxygen ability, eventually leading to respiratory and cardiovascular diseases^5–7^.

In recent years, many studies indicated that trees can significantly reduce PM_2.5_ and can absorb gaseous air contaminants^8–11^, especially at urban and suburban areas. Studies indicated that approximately 215000 t of total air PM_10_ were removed by urban trees in the United States^12^, and an increase in tree cover from 3.7% to 16.5% removed approximately 200 tons of PM_10_ each year in the West Midlands^13^. Forest canopies significantly altered the sulfur concentration and sedimentation rate of PM_2.5_ in a coniferous forest in central Japan and in a Norway spruce forest^14,15^. The ability of trees to clean the air might be related to the following: an increase in vegetation cover, which reduces the sources of PM_2.5_; PM can be absorbed by different tree organs; a decrease in wind speed may result in PM fallout; and changing wind direction might prevent PM_2.5_ transport into certain areas^16–19^. Various factors, e.g., the concentration of atmospheric PM_2.5_ and PM_10_, weather conditions, and tree biological characteristics, affect the ability of trees to remove PM_2.5_^16,20^. Researchers have focused mainly on broad-leaved and coniferous trees, such as spruce, cypress, pine, gingko, and crepe myrtle^21–23^, whereas very few studies have been conducted on bamboo. In addition, research on the mechanisms of plant ecological responses to haze is insufficient.

PM is a complex mixture of elemental carbon, organic carbon, ammonium elements, and water^24,25^. The chemical properties of PM are responsible for observed health effects. Polycyclic aromatic hydrocarbons (PAHs) account for a large fraction of the mass of PM, and they might be important in PM toxicity^26,27^, e.g., BaP was identified as an indicator of carcinogenic risk^28,29^. PAHs can be present in water^30,31^, soils^32^, and plants^33^. The distribution of PAH was closely related to the meteorological conditions. When the fog and haze came up, the concentration tended to increase^34^. High levels of atmospheric PAHs impose serious environmental and biological problems^28^. Some studies aimed to illustrate the role that forests play in regulating atmospheric PAHs and PM_2.5_ and their relationship. Previous studies stated that plants might regulate PM_2.5_ and PAHs through the absorption of PAHs by plants or the forest ecology system^33,35,36^. Further research conformed that trees play an important role in accumulating contaminations^37^, and the PAH level in leaves is considered a biomonitor of air pollution^38^. However, limited research has been conducted to illustrate the relationship between bamboo and PAHs.

Volatile organic compounds (VOCs) are important precursor substances for photochemical smog^39^ and seriously threaten human health as important atmospheric pollutants^40,41^. VOCs can damage the human body through respiratory passages, the digestive canal and skin, increasing the probability of mutation, deformity and cancer. In addition, VOCs are important precursors of tropospheric ozone photochemical generation: ozone, the peroxide group (PAN) and other photochemical oxidants are produced by reactions between VOCs and NO_x_, which simultaneously generate a range of free radicals, aldehydes, acids, and ketones, among other substances^42^. Changes in VOCs are strongly related to PM^43,44^ because photooxidation products of biogenic VOCs, mainly isoprene and monoterpenes, are significant sources of atmospheric PM in forested regions^42,45,46^. Thus, the role of VOCs in forest-regulated PM_2.5_ is an important research field.

*Phyllostachys edulis* is an important tree species with a high economic and ecologic value. The land area covered by *P. edulis* exceeds 4.43 million hm^2^ in China. And, many people focused on the developing of forest travel at lots of forest cities, such as Huzhou, Quzhou, Lishui, *et al*. at Zhejiang Province. These forest cities had one similar ecology factor that many forest land were grow at the suburban area. And *P. edulis* is one mainly plants that grow well at suburban area. Many previous researchers noted that that forest ecosystems have a significant effect on PM_2.5_ and PAHs^12,47^. However, few studies have focused on the relationship between bamboo forest and PM_2.5_, especially PAHs, an important component of PM_2.5_.

The aim of this study was to illustrate how *P. edulis* play a pivotal role in improving the air quality under solar and hazy day. Two types weather conditions were chosen to research the relationship between bamboo forest and air quality. The changes in atmospheric PM_2.5_, PAHs, and VOCs were observed in a typical bamboo forest. In addition, PAHs in bamboo leaves and soil were also measured to understand how pollutants enter into *P. edulis* forests. The following was hypothesized: (1) Bamboo forest can remove the atmospheric PM_2.5_ under hazy day; (2) Comparing on solar day, bamboo forest can absorb PAHs into leaves significantly on hazy day; and (3) On hazy day, components of atmospheric VOCs in bamboo forest changed.

## Results

### Changes in air quality on hazy and sunny days

#### Changes in atmospheric PM_2.5_ and PM_10_ concentration

The concentrations of PM_2.5_ and PM_10_ on the hazy day were significantly higher than those on the sunny day. On the hazy day, the concentrations of PM_2.5_ and PM_10_ ranged from 107.45–258.35 μg/m^3^ and 161.83–387.73 μg/m^3^, respectively, compared to those on the sunny day, which were stable at approximately to 8 μg/m^3^ and 40 μg/m^3^, respectively (Table 1). In addition, the ratio of PM_2.5_ to PM_10_ was significantly higher on the hazy day, i.e., over than 56% on hazy day, compared with 20% on the sunny day (Table 1).

**Table 1:** Changes in the atmospheric concentrations of PM_2.5_ and PM_10_ under different types of weather.

Weather	Time	Sites	PM_2.5_ (μg/m^3^)	PM_10_ (μg/m^3^)	PM_2.5_/PM_10_ (%)
Sunny day	All day	inside	8.49 ± 0.98a	38.06 ± 4.33b	22.30 ± 2.57a
		outside	8.61 ± 0.87a	42.80 ± 5.21a	20.34 ± 3.05ab
		edge	8.27 ± 0.92a	41.58 ± 6.61a	19.89 ± 3.98b
		P	30.66	<0.04	<0.001
Hazy day	Morning	inside	110.33 ± 2.43 g	161.83 ± 21.97e	0.75 ± 0.08a
		outside	120.58 ± 11.10 f	225.03 ± 10.98 cd	0.49 ± 0.03d
		edge	107.45 ± 2.87 g	210.73 ± 19.77d	0.51 ± 0.04d
	Noon	inside	162.65 ± 10.83d	274.90 ± 24.34c	0.59 ± 0.05c
		outside	171.22 ± 9.72c	253.38 ± 16.22c	0.68 ± 0.03ab
		edge	164.55 ± 17.39d	250.57 ± 30.70c	0.66 ± 0.09ab
	Afternoon	inside	140.62 ± 6.15e	252.43 ± 38.53c	0.56 ± 0.10 cd
		outside	146.12 ± 4.80e	242.00 ± 16.45c	0.60 ± 0.05c
		edge	148.05 ± 4.01e	259.22 ± 15.13c	0.57 ± 0.04 cd
	Night fall	inside	197.97 ± 10.98b	331.45 ± 28.75b	0.60 ± 0.07c
		outside	258.35 ± 30.61a	387.73 ± 38.20a	0.67 ± 0.02ab
		edge	197.00 ± 15.65b	319.18 ± 22.13b	0.62 ± 0.05bc
		P	<0.0001	<0.0001	<0.0001

Each value is the mean ± SE. Values followed by the same letter in the same column are not significantly different at the P > 0.05 level according to Tukey’s test. PM_2.5_, fine particulate matters with aerodynamic diameters less than 2.5 mm; PM_10_, fine particulate matters with aerodynamic diameters less than 10 mm; Inside, at the inside of *P. edulis* forest land; Outside, at the outside of *P. edulis* forest land; Edge, at the edge of *P. edulis* forest land.

The highest PM_2.5_ and PM_10_ were found at the nightfall time. The daily variations of PM_2.5_ and PM_10_ on the hazy day were greater than those on the sunny day and presented a trend of a slight increase followed by a sharp decrease and an increase to the highest concentrations at nightfall time, which reached 258.35 μg/m^3^ and 387.73 μg/m^3^, respectively, outside the forest (Table 1). By contrast, the PM_2.5_ and PM_10_ concentrations were lower than 170 μg/m^3^ and 275 μg/m^3^ at any other time, both inside and outside forest (Table 1).

The bamboo forest had a significant effect on the PM. The concentrations of PM_2.5_ and PM_10_ decreased significantly about 20% and 15%, respectively, inside the forest at nightfall time. In the morning and at night fall, the PM_10_ concentration in the interior of the forest was significantly lower than that outside the forest; no significant differences between the inside and outside of the forest were observed at any other time. At most times, the bamboo forest resulted in a decrease in the PM_2.5_ (Table 1). Thus, it can be inferred from this research that bamboo forests can buffer changes in PM_2.5_ and PM_10_ during the day time.

#### Changes in the atmospheric PAH content on hazy and sunny days

The total atmospheric content of the six main PAHs (T_air_) on the hazy day was significantly higher than that on the sunny day. The main atmospheric PAHs were four-, five- and six-ring compounds (BahA, BaP, BbF, BkF, Icdp and BghiP), which are known to be principal components of PAHs. T_air_ exceeded 12 ng·m^−3^ on the hazy day, compared with 1.04 ng·m^−3^ on the sunny day (Table 2). In addition, the concentrations of the main PAHs, such as BbF, BkF, BaP, BahA, IcdP, and BghiP, were also higher than those on the sunny day (Table 2).

**Table 2:** Atmospheric PAH concentrations at different areas (inside, outside and edge of the bamboo forest) (ng·m^−3^).

Weather	Sites	BbF	BkF	BaP	BahA	IcdP	BghiP	T_air_
Sunny day	inside	0.18 ± 0.02d	0.16 ± 0.02d	0.07 ± 0.01d	0.01 ± 0.00d	0.08 ± 0.00e	0.09 ± 0.00c	0.59 ± 0.03d
	outside	0.30 ± 0.02c	0.23 ± 0.01c	0.12 ± 0.01c	0.03 ± 0.00d	0.18 ± 0.00c	0.18 ± 0.04b	1.04 ± 0.01c
	edge	0.29 ± 0.11c	0.25 ± 0.05c	0.11 ± 0.03c	0.02 ± 0.00d	0.12 ± 0.00d	0.12 ± 0.01bc	0.91 ± 0.01c
Hazy day	inside	5.38 ± 0.66ab	1.90 ± 0.21b	0.67 ± 0.05b	0.32 ± 0.01b	3.24 ± 0.28a	1.40 ± 0.11a	12.91 ± 0.65b
	outside	6.10 ± 0.22a	2.25 ± 0.16a	0.80 ± 0.05a	0.38 ± 0.01a	3.34 ± 0.03a	1.46 ± 0.00a	14.44 ± 0.86a
	edge	5.18 ± 0.03b	1.86 ± 0.085b	0.68 ± 0.01b	0.20 ± 0.05c	3.05 ± 0.07b	1.30 ± 0.03a	12.27 ± 0.54b
	P	<0.0001	<0.0001	<0.0001	<0.0001	<0.0001	<0.0001	<0.0001

Each value is the mean ± SE. Values followed by the same letter in the same column are not significantly different at the P > 0.05 level according to Tukey’s test. BbF, Benzo b fluoranthrene; BkF, Benzo k fluoranthrene; BaP, benzo a pyrene; BahA, DiBenz (a,h) anthracene; IcdP, Indeno (1,2,3-c,d) pyrene; BghiP, Benzo (g,h,i) perylene; T_air_, the total content of mainly PAH in air; Inside, at the inside of *P. edulis* forest land; Outside, at the outside of *P. edulis* forest land; Edge, at the edge of *P. edulis* forest land.

The bamboo forest had a similar effect on the atmospheric concentration of PAHs on the hazy and sunny days. On both days, T_air_ inside the forest was significantly lower than that outside the forest. On the sunny day, the lowest T_air_ measured inside the forest was 0.59 ng·m^−3^. On the hazy day, T_air_ inside the forest was 12.91 ng·m^−3^, compared with 14.44 ng·m^−3^ outside the forest (Table 2).

These results indicate that this bamboo forest had a positive effect in regulating atmospheric PAHs on the hazy day, resulting in improved air quality.

#### Changes in the atmospheric concentration of VOCs on hazy and sunny days

On the hazy day, the atmospheric VOC content was significantly higher than that on the sunny day, and the 10 VOC compounds present in the highest concentrations differed significantly between the hazy and the sunny day. The atmospheric VOC content inside and outside the forest reached 94.77 and 156.85 µg/m^3^ on the hazy day (Table 3), compared with only 62.53 and 76.40 µg/m^3^, respectively, on the sunny day (Table 4).

**Table 3:** The 10 VOCs present in the highest concentration on the hazy day.

No.	VOCs	Inside (μg/m^3^)	Outside (μg/m^3^)	The ratio of inside to outside (%)
1	Butane, 2-methyl-	4.62 ± 0.23	9.93 ± 0.88	46.53
2	Propane, 2-methoxy-2-methyl-	11.53 ± 0.98	16.84 ± 1.43	68.47
3	Acetic acid	14.58 ± 1.13	*<0.01	/
4	Toluene	17.66 ± 1.43	31.82 ± 2.99	55.50
5	Benzene, 1,3-dimethyl-	4.84 ± 0.36	*6.83 ± 0.89	70.86
6	Benzaldehyde	2.75 ± 0.18	*4.96 ± 0.37	55.44
7	Acetophenone	7.31 ± 0.48	*5.78 ± 0.44	126.47
8	Nonanal	10.12 ± 0.96	11.34 ± 0.87	89.24
9	Decanal	11.35 ± 1.14	10.23 ± 1.14	110.95
10	Benzoic acid	10.01 ± 1.22	*1.52 ± 0.05	658.55
11	1,3-Butadiene, 2-methyl-	*3.72 ± 0.23	30.95 ± 2.76	12.02
12	Acetone	*<0.01	7.95 ± 0.69	/
13	Pentane, 2-methyl-	*1.23 ± 0.08	9.51 ± 0.87	12.93
14	n-Hexane	*1.81 ± 0.06	14.12 ± 1.28	12.82
15	Carbon Tetrachloride	*2.72 ± 0.11	14.16 ± 1.36	19.21
	T_VOCs_	94.77	156.85	60.42

The data marked with * was not in the list of 10 VOCs present in the highest concentration. Inside, at the inside of *P. edulis* forest land; Outside, at the outside of *P. edulis* forest land; T_VOCs_, total concentrations of the 10 VOCs present in the highest concentration.

**Table 4:** The 10 VOCs present in the highest concentration on the sunny day.

No.	VOCs	Inside (μg/m^3^)	Outside (μg/m^3^)	The ratio of inside to outside (%)
1	Ethyl Acetate	2.83 ± 0.31	3.23 ± 0.34	87.62
2	Benzene	4.02 ± 0.43	6.33 ± 0.76	63.51
3	Propane, 1,2-dichloro-	9.14 ± 0.88	14.01 ± 0.87	65.24
4	Toluene	25.36 ± 1.96	30.46 ± 2.37	83.26
5	Acetic acid, butyl ester	2.21 ± 0.27	2.73 ± 0.26	80.95
6	Formamide, N,N-dimethyl-	1.43 ± 0.09	*<0.01	/
7	Ethylbenzene	4.95 ± 0.32	5.62 ± 0.48	88.08
8	Benzene, 1,3-dimethyl-	5.83 ± 0.67	6.56 ± 0.86	88.87
9	o-Xylene	2.56 ± 0.18	2.95 ± 0.32	86.78
10	Benzaldehyde	2.67 ± 0.24	2.98 ± 0.26	89.60
11	Nonanal	*<0.01	1.53 ± 0.09	/
	T_VOCs_	62.53	76.40	81.85

The data marked with * was not in the list of 10 VOCs present in the highest concentration. Inside, at the inside of *P. edulis* forest land; Outside, at the outside of *P. edulis* forest land; T_VOCs_, total concentrations of the 10 VOCs present in the highest concentration.

More than 9 compounds, such as benzoic acid, acetone, and decanal, which were present in the list of 10 highest concentrations inside or outside the forest on the hazy day were not the same as those for the sunny day (Tables 3 and 4). This might be caused by the haze and was correlated with the increase in PM_2.5_ in atmospheric.

**Figure 1:**
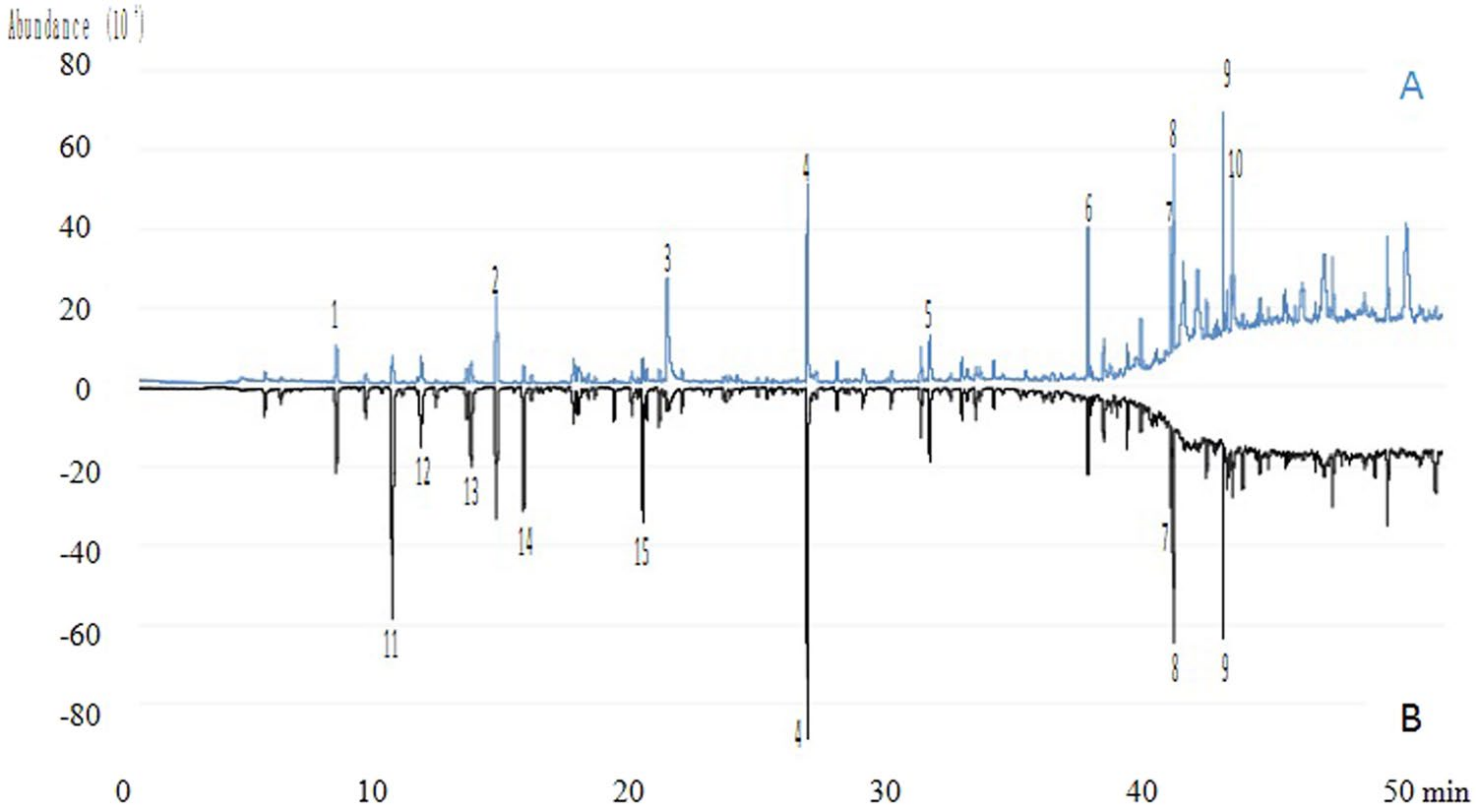
Total Ion Chromatogram of VOCs on hazy day. Part A, at the inside of *P. edulis* forest land; Part B, at the outside of *P. edulis* forest land; 1, Butane, 2-methyl-; 2, Propane, 2-methoxy-2-methyl-; 3, Acetic acid; 4, Toluene; 5, Benzene, 1,3-dimethyl-; 6, Benzaldehyde; 7, Acetophenone; 8, Nonanal; 9, Decanal; 10, Benzoic acid; 11, 1,3-Butadiene, 2-methyl-; 12, Acetone; 13, Pentane, 2-methyl-; 14, n-Hexane; 15, Carbon Tetrachloride. These VOCs were in the list of top 10 highest concentration compounds at the inside or outside of *P. edulis* forest land.

**Figure 2:**
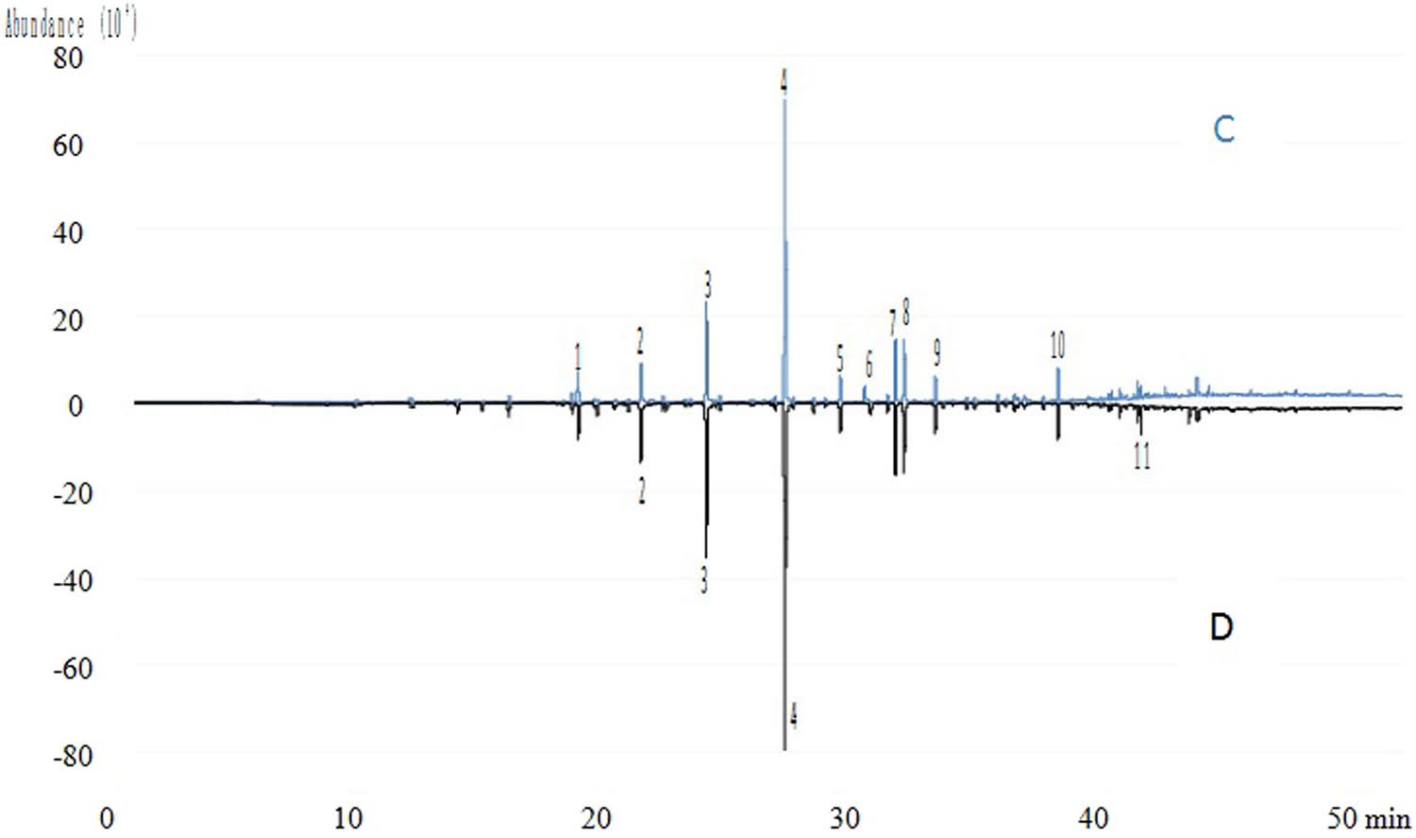
Total Ion Chromatogram of VOCs on sunny day. Part C, at the inside of *P. edulis* forest land; Part D, at the outside of *P. edulis* forest land; 1, Ethyl Acetate; 2, Benzene; 3, Propane, 1,2-dichloro-; 4, Toluene; 5, Acetic acid, butyl ester; 6, Formamide, N,N-dimethyl-; 7, Ethylbenzene; 8, Benzene, 1,3-dimethyl-; 9, o-Xylene; 10, Benzaldehyde; 11, Nonanal. These VOCs were in the list of top 10 highest concentration compounds at the inside or outside of *P. edulis* forest land.

The bamboo forest resulted in a decrease in the VOC content on both hazy and sunny days. On the hazy day, the concentration of VOCs inside the forest was 39.58% lower than that outside the forest, and half of the VOCs present in the highest concentrations differed between the inside and outside of the forest (Fig. 1). On the sunny day, the concentration of VOCs inside the forest was 18.15% lower than that outside the forest, and most of the compounds present in the highest concentrations were the same inside and outside the forest (Fig. 2). This indicated that the bamboo forest played a positive role in regulating atmospheric VOCs.

### Changes in the PAHs concentrations in leaves on hazy and sunny days

The total concentrations of the six main PAHs in leaves (T_leaf_) were significantly higher on the hazy day. T_leaf_ inside the forest and at the forest edge was higher on the hazy than on the sunny day, by approximately 110% and 60%, respectively (Table 5). Further analysis indicated that the concentrations of most compounds (besides BkF and Bap) increased rapidly on the hazy compared with the sunny day.

**Table 5:** PAH concentrations in bamboo leaves in different areas (inside and edge of the bamboo forest) (μg/kg).

Weather	Sites	BbF	BkF	BaP	BahA	IcdP	BghiP	T_leaves_
Sunny day	inside	25.25 ± 0.07b	21.90 ± 0.57a	31.45 ± 2.12a	2.30 ± 0.00b	6.04 ± 0.03c	0.05 ± 0.00d	86.99 ± 2.35b
	edge	4.84 ± 0.08c	5.18 ± 0.08c	30.95 ± 3.54a	0.70 ± 0.01c	3.62 ± 0.06d	0.11 ± 0.00c	45.40 ± 2.47c
Hazy day	inside	41.51 ± 4.58a	11.67 ± 0.67b	32.22 ± 0.5a	4.40 ± 0.39a	60.04 ± 3.01a	32.51 ± 2.39a	182.35 ± 8.64a
	edge	21.85 ± 0.59b	5.72 ± 0.06c	19.72 ± 4.42b	0.49 ± 0.03c	16.64 ± 0.65b	8.76 ± 0.3b	73.18 ± 6.32b
	P	<0.0001	<0.0001	<0.0001	<0.0001	<0.0001	<0.0001	<0.0001

Each value is the mean ± SE. Values followed by the same letter in the same column are not significantly different at the P > 0.05 level according to Tukey’s test. BbF, Benzo b fluoranthrene; BkF, Benzo k fluoranthrene; BaP, benzo a pyrene; BahA, DiBenz (a,h) anthracene; IcdP, Indeno (1,2,3-c,d) pyrene; BghiP, Benzo (g,h,i) perylene; T_leaves_, the total content of mainly PAH in leaves; Inside, at the inside of *P. edulis* forest land; Edge, at the edge of *P. edulis* forest land.

On both the hazy and sunny days, T_leaf_ inside the forest was significantly higher than that at the edge of the forest and reached 182.35 and 86.99 μg/kg, respectively (Table 3). On the hazy day, T_leaf_ inside the forest was 130% higher than that at the edge of the forest. Most of the compounds exhibited similar trends.

It can be deduced that the bamboo forest had a positive effect in reducing atmospheric PAHs. On the hazy day, the increase in the PAH concentration of the leaves was correlated with the increase in outside forest atmospheric PAHs (Table 5), especially the leaves inside the bamboo forest; the PAH concentration decreased after a long time. In this study, the sunny day occurred later in the year than the hazy day, and the concentration of PAHs in leaves decreased, both inside and at the edge of the forest (Table 5). It can be inferred from this study that bamboo leaves can absorb some atmospheric PAHs, especially those inside in the forest. In addition, some of the PAHs absorbed by leaves may be transferred to other bamboo organs, water, or soil.

### Changes in the concentrations of PAHs in soil on hazy and sunny days

On the hazy day, the total concentrations of the six main PAHs (T_soil_) in soil were significantly higher than those on the sunny day. T_soil_ inside and at the edge of the forest on the hazy day was higher than that on the sunny day, by approximately 235% and 70%, respectively. In addition, the concentrations of all six compounds were higher on the hazy than on the sunny day (Table 6).

**Table 6:** PAH concentrations in soil in different areas (inside and edge of the bamboo forest) (μg/kg).

Weather	Sites	BbF	BkF	BaP	BahA	IcdP	BghiP	T_soil_
Sunny day	inside	18.54 ± 1.81c	6.05 ± 0.07c	6.16 ± 0.08c	0.88 ± 0.06c	8.65 ± 0.10c	6.30 ± 0.55c	46.58 ± 3.76c
	edge	16.24 ± 2.50c	4.30 ± 0.56d	4.96 ± 0.92d	0.82 ± 0.16c	9.60 ± 1.94c	4.76 ± 0.85d	40.67 ± 4.02c
Hazy day	inside	79.8 ± 1.34a	54.7 ± 1.27ba	34.55 ± 1.20a	8.69 ± 0.07a	32.1 ± 1.13a	27.05 ± 0.35a	236.89 ± 18.87a
	edge	23.2 ± 0.76b	17.7 ± 0.95b	8.45 ± 0.13b	1.97 ± 0.10b	12.62 ± 1.08b	8.24 ± 0.10b	72.18 ± 5.16b
	P	<0.0001	<0.0001	<0.0001	<0.0001	<0.0001	<0.0001	<0.0001

Each value is the mean ± SE. Values followed by the same letter in the same column are not significantly different at the P > 0.05 level according to Tukey’s test. BbF, Benzo b fluoranthrene; BkF, Benzo k fluoranthrene; BaP, benzo a pyrene; BahA, DiBenz (a,h) anthracene; IcdP, Indeno (1,2,3-c,d) pyrene; BghiP, Benzo (g,h,i) perylene; T_soil_, the total content of mainly PAH in soils; Inside, at the inside of *P. edulis* forest land; Edge, at the edge of *P. edulis* forest land.

The bamboo forest also had an important effect on the PAH content of the soil on the hazy day. Specifically, the concentrations of all 6 PAH compounds in the soil inside the forest were higher than those at the edge of the forest on the hazy day; however, significant differences were only found for BkF, BaP and BghiP between the inside and edge of the forest.

## Discussion

Although obvious bodily harm results from increasing PM_2.5_ concentrations, it is difficult to completely eliminate the production of PM_2.5_ from different sources in China due to the rapid rate of economic development of this country. Therefore, it is important that research is conducted on how to remove atmospheric PM_2.5_ and lower the concentrations of other atmospheric pollutants. Many studies have been conducted on the potential of trees as a mitigation tool for atmospheric particles. Forests have a significant positive effect on the environment through a reduction in pollution, or they directly affect PM in the atmosphere by removing particles^13,48^. Trees have been shown to significantly reduce atmospheric PM^12,49^, and an increase in tree vegetation can also help to remove PM^13^. In this study, the atmospheric PM concentrations were significantly decreased by the *P. edulis* forest. This might be related to the fact that *P. edulis* is an evergreen species that can reproduce and propagate through rhizomes. Previous studies showed that evergreen species had a greater ability to reduce PM than deciduous trees^50^, because the leaves of evergreen species persist year-round, especially in winter and spring when hazy fog occurs frequently, resulting in a greater reduction in PM^17,51^. In the present study, the PM_10_ concentration inside the forest was higher than that outside the forest at several times. This might be related to meteorological factors (e.g., temperature and humidity) in the early morning being different to those at other times, and this can affect the ability of trees to remove PM^18^. And trees may have a lower CO_2_ assimilation rate, which can also affect PM absorption in a forest^51^.

PAHs, a class of hydrocarbons that threaten human health, bind to PM_2.5_^34,52–54^. As a large fraction of the mass of PM, are candidate components with respect to PM toxicity. In this study, the atmosphere contained a large amount of carcinogenic and mutagenic PAHs on the hazy day; BbF, BkF and BaP (5-ring compounds), followed by those with 4 and 6 rings, i.e., BahA, BghiP, IcdP, BbkF (BbF and BkF), were dominant in all TSP samples, which is consistent with reports for GuiYu^54^ and Hong Kong^34^. PAHs are removed by trees at the same time as PM_2.5_, and the decrease of PM_2.5_ might be a result of plant capture and retention into the bamboo forest leaves and soil. On the one hand, plant decreased PM_2.5_ concentration by capture PM in air. Previous documents showed that PM can be intercepted by plant organs, such as leaves, bark, and twigs, resulting in the removal of PM_2.5_. The intercepted particles can be absorbed into the tree, though most particles that are intercepted are retained on the plant surface. The ability to intercept PM_2.5_ varies with tree species^48,55^, roughness of leaves^56,57^, cilia number on leaves^58,59^, pore structure^11^, wax coat^60^, and environmental conditions^16,49^. Studies have indicated that PAHs are absorbed together with PM_2.5_ absorption^33,56,61^. This result is consistent with the findings of this study. In this study, atmospheric PAHs decreased and PAHs in bamboo leaves were significantly increased. The total concentrations of the six main PAHs in leaves (T_leaf_) were significantly higher on the hazy day. On the other hand, retention by forests is an important way to reduce atmospheric PM_2.5_. Trees have the capacity to retain PM^21,62^. In this study, PAHs, an important component of PM_2.5_, accumulated significantly in soil on the hazy day. Researchers have reported that indicator matter in forest soils were significantly higher than in non-forest land^15,63^. Forests alter the sedimentation rate of PM_2.5_ and increase the rate at which PM infiltrates the soil. According to radioactive substances or the indicator substance tracking technique, PM in forests was significantly higher than that in non-forest land^19,64^. Studies conducted in coniferous forests of central Japan and in Norway spruce forests also indicated that the forest canopy significantly altered the sulfur concentration and sedimentation rate of PM_2.5_^14,19^.

The effect of forest on air quality was more obvious under hazy weather condition. The effects of weather conditions on PM were very significant^18,65,66^.The wind speed affected the horizontal diffusion of aerosols. The temperature rise was conducive to aerosol diffusion and was also beneficial to secondary aerosol production. Humidity would cause ultra-fine aerosols to aggregate^65^. In the forest, the temperature increased, the vertical convection in the atmosphere increased, and the concentration of PM10 and PM2.5 in the forest belt would be reduced. The concentration of the relative humidity increased the concentrations of PM10 and PM2.5^66^. Under the foggy conditions, the general temperature was lower and the wind speed was smaller. The droplets that make up the fog were suspended in the atmosphere near the ground layer. It was very easy to absorb the polluted particles in the air, which affected the distribution of organic pollutants in the atmosphere. The daily average concentration of PAHs monomer was significantly higher than that of sunny days, and maintained high concentration throughout the day and night^34^. When the air quality in foggy weather was particularly poor, it was easy to form haze. The level of PAHs in leaves was promoted under hazy day^56^. And there were many problems were found in plant materials being contaminated by PAHs^67,68^. For the exposure to pollution, such as PAH pollutants, the leaves’ surface and structure changed^69^, e.g PM was found in stomata^70^. And the leaves surface was more easily to bacterial and fungal infections. These changes caused high ability to retain water, and means that PAHs may affect the amount of retained rainfall indirectly^37^. Thus, the hazy day affected the plant materials, and plant leaves showed more ability to capture pollutants. These were in consisting to our study, that bamboo showed significant ability to remove PM2.5 and PAHs, and the PAHs in leaves increased significantly under hazy day.

VOCs are strongly related to PM_2.5_ because photochemical oxidation and ozonolysis of monoterpenes can lead to secondary organic aerosol (SOA) formation^71,72^. Photooxidation products of biogenic VOCs, mainly isoprene and monoterpenes, are significant sources of atmospheric PM in forested regions^42^. In this study, the concentrations of atmospheric VOCs were significantly different on the hazy and the sunny day, especially the 10 VOCs present in the highest concentrations. This might be because the air pollution had different sources of VOCs on the sunny and the hazy day. Changes in the sources of VOCs affect the components of VOCs^39^. This also indicated that the VOCs and PM_2.5_ were important factors contributing to the hazy day. Furthermore, atmospheric VOCs can be significantly regulated by plants^40^. The changes in atmospheric VOCs in this study might be attributed to changes in the weather condition or because the bamboo forest affects the components of VOCs. Vegetation releases numerous VOCs into the atmosphere, particularly isoprene, monoterpenes, and sesquiterpenes, as well as a series of oxygen containing compounds^41^. In addition, isoprene can also result in SOA, including species such as 2-methyltetrols (2-methylthreitol and 2-methylerythritol), C_5_-alkene triols (cis- and trans-2-methyl-1,3,4-trihydroxy-1-butene and 3-methyl-2,3,4-trihydroxy-1-butene) and 2-methylglyceric acid^42,73^. Isoprene SOA products have been detected at various forested sites around the world^74,75^, which is similar to this study because several substances were also observed in the bamboo forest. In addition, atmospheric VOC distribution might be affected by changes in the environment, as well as the plant canopy, which can change the wind speed, temperature, and humidity, among other factors^76^. This indicated that the bamboo forest regulated the VOCs to adapt to the polluted environment.

## Materials and Methods

### Experimental design

A *P. edulis* forest in Changxing, Zhe Jiang, was selected as the investigation object. Sampling was performed on a mountain in one of the countries of Changxing in the southeastern region of Zhejiang Province (30°43′–31°11′N, 119°33′–20°06′E). The climate of Changxing is classified as subtropical monsoon maritime, with an annual average temperature of 15.6 °C and annual precipitation of 1309 mm. The forest is a plantation land with a density about 180–210 plants/667 m^2^ and 12–15 m height. The land is far away from the nearest town about 7.2 km. And the 3–5 years old bamboo individuals were respect for about 70%.

The study consisted of 2 weather types (sunny day, hazy day) and 3 sites (interior of the bamboo forest, the edge of the bamboo forest, and outside the bamboo forest). There were 3 bamboo forest land were selected as 3 replications. Atmospheric PM_2.5_, PM_10_, PAH and VOC concentrations were measured at 3 sites. The PAH concentrations in both bamboo leaves and soil were analyzed at 2 sites (interior and the outside of the bamboo forest). The hazy day treatment was selected at a time when the haze had persisted for more than one month and the PM_2.5_ concentration exceeded 200 µg•m^−3^. The sunny day was selected at a time when the weather was continuously fine for more than one week. The hazy day was considered as air pollution treatment, and the sunny day was considered as control treatment. This work is guided on “Observation Methodology for Long-term Forest Ecosystem Research of  National Standards of the People's Republic of China (GB/T 33027-2016).

### Sampling method

#### Method used for air sample collection

The method for air sample collection was based on the industrial or national standard that focused on the monitoring of air or particulate quantity^77,78^. Medium-flow air samplers (Wuhan Tianhong Instrument Limited Liability Company, Wuhan, China) were used to collect samples with a flow rate of 100 L/min for PAH analysis and a flow rate of 0.5 L/min for VOC analysis. The ambient air samples were collected from the atmosphere at heights of approximately 1.5 m above ground.

Before sampling, filters were conditioned at 25 °C and 40% relative humidity in a desiccator for at least 24 h. PAH-associated contaminants were isolated from the atmosphere by drawing air through a Whatman quartz fiber filter (QFF, 800–1000). VOC-associated contaminants were isolated from the atmosphere by drawing air through a Whatman quartz fiber filter for approximately 30 min. Background contamination was monitored by using operational blanks, which were processed simultaneously with the samples. After sampling, the filters were wrapped in aluminum foil and stored in ziplock bags at −20 °C.

#### Methods for leaf and soil sample collection

Soil and leaf samples were collected according national or industrial standards that are used to monitor the environment^79,80^. At every forest land, the bamboo leaves were selected on the branches at about 3 m height in 4 directions, and 10 leaves were collected every direction. These were done 3 replications and samples gather into an ice bag. Then the samples were taken to the laboratory to processing.

The surface soil samples (0–20 cm) were collected with quartering division method at every bamboo land. 3 replicate samples collected from one land were mixed uniformly, respectively. The soil samples were ground with a pestle and mortar, screened through an 80-mesh sieve, and stored in a mason jar for the determination of PAHs and VOCs.

### Methods for detection and analysis

#### Method for PM_2.5_ and PM_10_ concentration detection

PM_2.5_ and PM_10_ concentrations were detected using a dust detector (DUSMATE) according to the industrial standard^77,81^. The instrument was adjusted to the on-line monitoring system before detection. PM_2.5_ and PM_10_ concentrations were detected every 3 h at a height of 1.5 m during the monitoring period.

#### Extraction and analysis of PAHs

PAH analysis was performed using the GC-MC method, and the analysis details were provided by the relative industrial standard for the quantification of air and particulate material^77^ and elsewhere^54^. Briefly, the filter samples from ambient air, soil and bamboo leaves were repeatedly reflux extracted using a soxhlet extractor with aether:hexane (1:9) for at least 16 h, no less than 4 times per hour. Anhydrous sodium sulfate (15 g) was added to the extract to ensure free flow of sodium sulfate particles. The extracts were then concentrated to 5.0 ml using rotary evaporation. Subsequently, hexane (5–10 ml) was added and rotary evaporated until less than 1 ml hexane remained. To prevent interference, extracts were purified using silica gel chromatography. Extracts were analyzed for PAHs by gas chromatography mass spectrometry using Agilent’s New 7890B Gas Chromatograph and a 5977 A Series Mass Selective Detector (GC-MSD) operated in the full ion scanning mode.

#### Analysis of VOCs

VOCs were analyzed using the industrial standard methods for air and particulate monitoring^77,78^. The VOC concentration was measured using a thermal desorbed instrument (Tekmar 6000/6016) interfaced with a gas chromatograph (HP 7890 B) and mass selective detector (HP 5977 A, AMA Co., Germany). The working conditions of the TDS were as follows: gas pressure of 20 kPa, inlet temperature of 250 °C desorption temperature of 250 °C for 10 min; cold trap temperature held at 120 C for 3 min, followed by a rapid increase to 260 °C. An HP-5MS column (50 m, i.d. 0.25 mm, and film thickness 0.25 m) was used for chromatographic separation. The temperature program was 40 °C for 3 min followed by 10 °C/min up to 250 °C for 3 min and then an increase to 270 °C. The ion energy of the MS (type 5975 C, Agilent) was 70 eV; the ion source temperature was 230 °C; the Quadrupole temperature and the interface temperature were 150 and 280 °C, respectively; and the mass spectrometry scanning mass ranged from 28 to 450 m/z. The retrieval and qualitative analysis of the mass spectrometry data were accomplished using the NIST 2008 library, which was housed in the computer of the temperature instrument. In addition, the chromatographic peak area normalization method was used for the calculation of the relative concentrations of the mass spectrometry data.

### Statistical Analysis

Analysis of variance and Duncan’s new multiple range tests were performed with SAS 9.2 Institute Inc.™ (1999) software. The data are presented as means ± S.D. Differences at P < 0.05 were considered significant.

### Availability of materials and data

The datasets generated during and analyzed during the current study are available from the corresponding author on reasonable request.

## Conclusion

Bamboo forest shows strong effects on reduction of the air pollution. The concentrations of atmospheric PM_2.5_, PM_10_, PAHs and VOCs decreased at inside of forest land. And by tracing the increase of PAHs in bamboo leaves and soils, the PM_2.5_ might be cleaned by plant capture and bamboo forest renitent. These can illustrate that bamboo forest remove PM_2.5_ by gathering PAHs into the forest ecosystem factors, e.g. bamboo leaves and soils. And bamboo forest also changed the VOCs concentration in air, and also changed the types of 10 VOCs present in the highest concentration list inside and outside forest land, to affect PM_2.5_. PAHs were important substances to explain the PM_2.5_ regulate way. These findings illustrated a regulating way for *P. edulis* under haze day, and were help to understand its ecology value, especially the role of bamboo forest played in providing ecosystem services at urban or suburban. These also indicated that it is essential to start the research on the effect of *P. edulis* forest under different plantation types, different areas, and evenly the effect of other bamboo species. Also, these findings demonstrated that it was also essential to research the responses of bamboo to ecological factors, especially the polluted air, to understand the biological and physiological mechanism. And many physiological ecology study methods, such as isotopic tracing, confocal laser scanning microscope, manual simulation, *et al*., might help to do it well.
